# Evaluation of ^¹¹¹^In-Labelled Exendin-4 Derivatives Containing Different Meprin β-Specific Cleavable Linkers

**DOI:** 10.1371/journal.pone.0123443

**Published:** 2015-04-09

**Authors:** Andreas Jodal, Fabienne Pape, Christoph Becker-Pauly, Ole Maas, Roger Schibli, Martin Béhé

**Affiliations:** 1 Center for Radiopharmaceutical Sciences ETH-PSI-USZ, Paul Scherrer Institute, Villigen, Switzerland; 2 Department of Chemistry and Applied Biosciences, ETH Zurich, Zurich, Switzerland; 3 Institute of Biochemistry, University of Kiel, Kiel, Germany; 4 Department of Radiology and Nuclear Medicine, Division of Nuclear Medicine, University Hospital Basel, Basel, Switzerland; J. Heyrovsky Institute of Physical Chemistry, CZECH REPUBLIC

## Abstract

**Background:**

Cleavable linkers, which are specifically cleaved by defined conditions or enzymes, are powerful tools that can be used for various purposes. Amongst other things, they have been successfully used to deliver toxic payloads as prodrugs into target tissues. In this work novel linker sequences targeting meprin β, a metalloprotease expressed in the kidney brush-border membrane, were designed and included in the sequence of three radiolabelled exendin-4 derivatives. As radiolabelled exendin-4 derivatives strongly accumulate in the kidneys, we hypothesised that specific cleavage of the radiolabelled moiety at the kidney brush-border membrane would allow easier excretion of the activity into the urine and therefore improve the pharmacological properties of the peptide.

**Results:**

The insertion of a cleavable linker did not negatively influence the *in vitro* properties of the peptides. They showed a good affinity to the GLP-1 receptor expressed in CHL cells, a high internalisation and sufficiently high stability in fresh human blood plasma. *In vitro* digestion with recombinant meprin β rapidly metabolised the corresponding linker sequences. After 60 min the majority of the corresponding peptides were digested and at the same time the anticipated fragments were formed. The peptides were also quickly metabolised in CD1 nu/nu mouse kidney homogenates. Immunofluorescence staining of meprin β in kidney sections confirmed the expression of the protease in the kidney brush-border membrane. Biodistribution in GLP-1 receptor positive tumour-xenograft bearing mice revealed high specific uptake of the ^111^In-labelled tracers in receptor positive tissue. Accumulation in the kidneys, however, was still high and comparable to the lead compound ^111^In-Ex4NOD40.

**Conclusion:**

In conclusion, we show that the concept of cleavable linkers specific for meprin β is feasible, as the peptides are rapidly cleaved by the enzyme while retaining their biological properties.

## Introduction

Over recent years cleavable linkers targeting specific physiologic environments or enzymes have proven to be a versatile tool for various medical applications. Cleavable linkers are successfully used to reduce the side effects of toxic drugs, for example when camptothecin is conjugated to the carrying molecule substance P via a cleavable linker, it acts as a prodrug and is not toxic. Upon reaching its target, however, the linker is specifically cleaved and releases its cytotoxic payload into the desired tissue. This concept significantly improves the specificity of the drug and thereby minimises off-target side-effects [1,2]. Cleavable linkers are also used diagnostically in a new approach to identify diseases such as cancer or inflammation. New near infrared (NIR) probes, highly sensitive tools for the diagnosis of such conditions, have two fluorescent, self-quenching dyes attached to the probe via a specific self-immolative linker. This linker is cleaved in the targeted tissue, thereby liberating the dye and resulting in a specific fluorescent signal [3,4].

Nuclear medicine is another field that could benefit from the use of cleavable linkers. Accumulation of radioactivity in non-target tissues can both increase the background signal in diagnostic purposes, as well as potentially damage sensitive tissues in therapeutic approaches. Tracer containing linker sequences that are degradable by enzymes could reduce the unwanted accumulation and increase the specificity of the signal.

Many hydrophilic radiolabelled compounds with a molecular weight below 60 kD are excreted by the kidneys [5]. These radiopharmaceuticals are often reabsorbed in the proximal tubuli in the kidneys, resulting in a strong accumulation that can hinder diagnostic imaging. Additionally, as they are organs sensitive to radiation, a high radiation dose to the kidneys can cause permanent damage, potentially leading to future complications after radiotherapeutic interventions [6]. Kidneys are the dose-limiting organs in several nuclear medicine therapies [7]. Cleavable linkers located shortly before the radiolabelled moiety that are degradable by enzymes specifically expressed in the kidney brush border membrane would allow the easy cleavage and excretion of the radioactive metabolites into the urine. This concept was previously demonstrated when the introduction of a Gly-Lys linker into radiolabelled antibody fragments significantly reduced kidney accumulation [8,9]. The transfer to peptides, however, was not successful meaning that targeting different enzymes is necessary [10]. In a recently published study a new cleavable linker was proposed. ^67^NOTA-MI-Fab was shown to release ^67^Ga-NOTA-Met upon proteolysis in the lysosomes of the brush-border membrane, however, the structure of the radiometabolites and the cleavable linkages are not yet completely understood [11].

**Fig 1:**
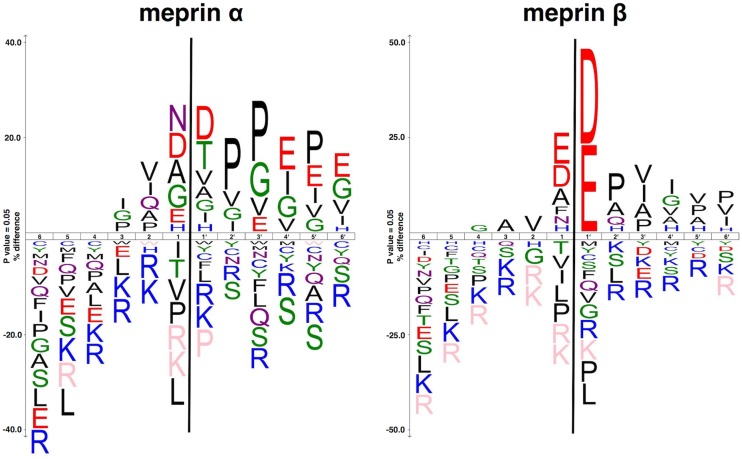
The most common amino acids around the cleavage sites of both meprin α and β. The size of the one letter code of the amino acid represents the frequency of that amino acid in that particular position. The figure was generated using icelogo, based on peptide cleavage assays described previously [13]. Peptide sequences were aligned on the scissile bond between P1 and P1′ indicated by a black line. Statistically significant amino acid residue occurrences present (P<0.05) were plotted. Those amino acids that were completely absent are shown below in pink.

Meprin α and meprin β, two astacin metalloproteases strongly expressed on the brush border membranes of kidney proximal tubular cells, could be potentially utilised to cleave linkers [12]. Meprin β, in contrast to meprin α, has a stronger preference for negatively charged amino acid residues around the scissile bond as determined by proteomics (Fig 1) [13]. The unique specificity of meprin β makes the design of specific linker sequences easier. Various substrates of meprins have been identified, almost all of them confirming the cleavage specificity [14,15]. Among the substrates of meprin β are gastric peptides such as cholecystokinine 8 and gastrin that share anionic amino acids around the cleavage site [16]. While most of those peptides are degraded, meprin β can also perform other functions like activating cytokines, as in the case of the processing of interleukin-1β precursor [10,17]. It has also been shown that meprins are expressed in a number of pathological conditions including cancer and fibrotic diseases, making these interesting targets for prodrugs with meprin-specific cleavable linkers [18–20].

Introducing a linker into the amino acid sequence of a peptide, however, is challenging. The insertion of an additional sequence into a peptide might influence its secondary structure and potentially lead to reduced affinity and altered pharmacokinetic properties. Our model substance, the 39 amino acid peptide exendin-4, is an agonist for the glucagon-like peptide 1 receptor (GPL-1R) specifically expressed on β-cells. These cells are located in the islets of Langerhans in the pancreas, where their activation mainly affects insulin secretion, insulin synthesis and β-cell proliferation [21]. Based on known substrates we designed three derivatives of exendin-4 to evaluate the feasibility of cleavable linkers as substrates for meprin β.

Pathologically altered β-cells can lead to imbalanced glucose levels with potentially severe complications. Both apoptosis of β-cells and their decreased sensitivity towards insulin can lead to diabetes and result in hyperglycaemia, while an increased function caused by either insulin-producing tumours (e.g. insulinoma) or by hyperplastic β-cells (e.g. nesidioblastosis) may lead to hypoglycaemia. In the latter cases particularly, accurate localisation is crucial to surgically remove those *foci*, currently the only curative treatment option for these conditions. ^111^In-labelled exendin-4 derivatives have been successfully tested in clinical trials for detection of insulinoma and to evaluate the β-cell mass of type 1 diabetic patients and transplanted β-cells. One remaining problem, however, is the high accumulation in the kidneys obstructing the view on the pancreas [22–24].

In this work we describe the characterisation of three peptides based on exendin-4 that contain a cleavable linker between the binding moiety and the In-labelled chelator that were designed as cleavable substrates for meprin β.

The linker of PSI-CLNOD1 is based on a sequence that can be also found within the binding sequence of exendin-4 spanning from Q13 to V19, which we have shown to be cleaved by meprin β. PSI-CLNOD2 contains a linker based on cholecystokinine-8 (CCK-8), a very good natural substrate for meprin β. Lastly, the cleavable linker of PSI-CLNOD3 was based on the three most frequent amino acids around the cleavage site of the protease [13]. We determined the binding properties of these peptides on Chinese hamster lung (CHL) cells expressing the GLP-1R as well as the internalisation kinetics. Further, we tested the stability of the probes. We assessed their metabolic stability in fresh human blood plasma as well as the feasibility of the linkers in both kidney homogenates and with recombinant meprin β. To verify the expression of meprin β in mouse kidneys CD1 kidney sections were stained with meprin β-specific immunoflourescent antibody. The biodistribution of the ^111^In-labelled derivatives was performed in CD1 nude mice bearing a CHL-GLP-1R positive tumour xenograft.

## Material and Methods

### Radiolabelling of the exendin-4 derivatives

**Fig 2:**
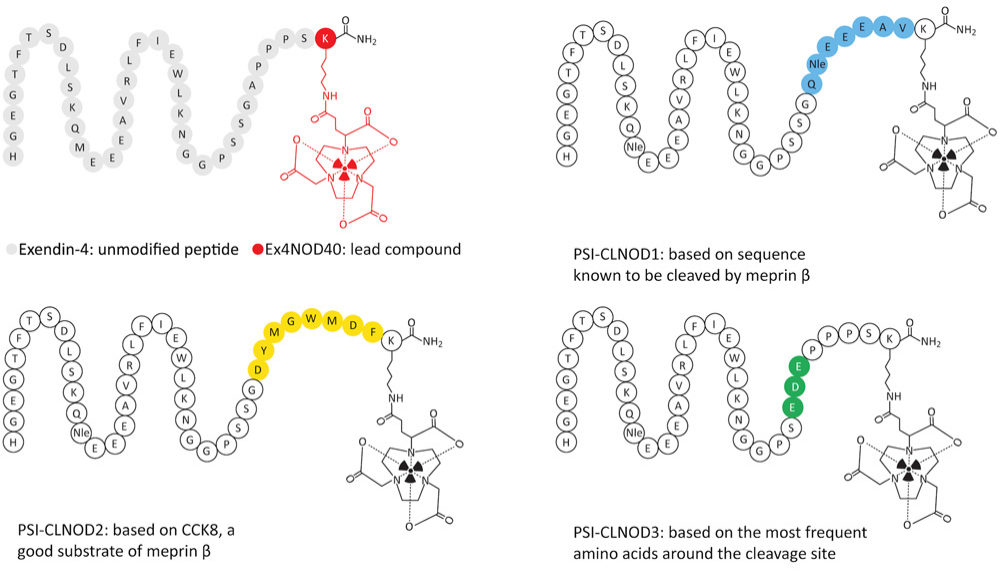
Graphical representation of exendin-4 and its derivatives. The linkers are highlighted in different colours.

Ex4NOD40, PSI-CLNOD1, PSI-CLNOD2 and PSI-CLNOD3, as seen in Fig 2 and S1 Table, were synthesised by Peptide Specialty Laboratories (Heidelberg, Germany). The fragments of the peptides, seen in Table 1, were synthesised by piCHEM (Graz, Austria). The chelator 1-(1,3-carboxypropyl)-1,4,7-triazacyclononane-4,7-diacetic acid (NODAGA) was attached to the ε-amino group of the amidated lysine at the C-terminal end of the peptide. Unmodified exendin-4 was purchased from Bachem (Bubendorf, Switzerland).

**Table 1:** Amino acid sequences of the peptide fragments.

**CL1-F1**	EEAVK(NODAGA)-NH_2_
**CL1-F2**	EAVK(NODAGA)-NH_2_
**CL1-F3**	K(NODAGA)-NH_2_
**CL2-F1**	DYMGWMDFK(NODAGA)-NH_2_
**CL2-F2**	DFK(NODAGA)-NH_2_
**CL3-F1**	DEPPPSK(NODAGA)-NH_2_
**CL3-F2**	EPPPSK(NODAGA)-NH_2_

All peptides were labelled with ^111^InCL_3_ (Mallinckrodt, Netherlands) by adding 9.4 MBq activity to 25 μL 0.02 M HCl and 0.23 to 0.25 nmol of the respective compound and 5 μL 0.5 M ammonium acetate (pH 5.5). Following incubation at 95°C for 30 min the quality of the labelling was assessed using reversed-phase high-performance liquid chromatography (RP-HPLC) on a C18 reversed-phase column (Dr. Maisch Reprospher 300 C18-TN, 4.6 mm x 150 mm; 5 μm). Before injection the sample was diluted in 0.1 mM sodium DTPA and eluted with water containing 0.1% TFA (trifluoroacetic acid) with a linear gradient from 15% to 95% acetonitrile for 10 minutes followed by an isocratic elution at 95% acetonitrile for an additional 5 min with a flow rate of 1 mL/min. For the *in vitro* meprin β digestion 10 nmol of the respective peptide were labelled as described above. After the labelling the remaining unlabelled chelators were saturated with ^nat^InCl_3_ (10 nmol, 1eq.) and once again incubated for 20 min at 95°C.

### Cell culture

Chinese hamster lung cell line stably transfected with the GLP-1 receptor gene (CHL-GLP-1R positive cells), a kind gift of Prof. Brigitte Lankat-Buttgereit, were cultured in Dulbecco’s Modified Eagle’s Medium (DMEM) with 4.5 g/L D-glucose and GlutaMax. In addition, the media contained 10% fetal calf serum, 100 IU/mL penicillin G, 10 mg/mL streptomycine, 500 μg/mL geneticin sulfate, 1 mM sodium pyruvate and 0.1 mM non-essential amino acids. The cells were maintained in a humidified 5% CO_2_ atmosphere at 37°C and were harvested by trypsinisation with trypsin/EDTA [25].

### Labelling of exendin-4 derivatives with ^nat^InCl_3_

Peptides were labelled by adding 10 nmol of the respective peptide to 20 nmol ^nat^InCl_3_ (Sigma-Aldrich, Switzerland) solution in 60 μL 0.4 M ammonium acetate buffer (pH 5.5) and subsequent incubation for 15 min at 90°C. The labelling was verified by liquid chromatography–mass spectrometry (LC/MS) on an Atlantis C18 (25 cm x 4.6 mm; 5 μm) column.

### IC_50_ binding assay

The experiments were conducted as described previously [26]. The half-maximal inhibitory concentrations (IC_50_) of ^nat^In-labelled PSI-CLNOD1, PSI-CLNOD2 and PSI-CLNOD3 were determined using CHL-GLP-1R positive cells. 4 kBq (0.1–0.6 pmol) ^111^In-labelled Ex4NOD40 was used for detection of the binding. The cells were treated with ^111^In-Ex4NOD40 and various concentrations of either ^nat^In-labelled exendin-4 derivative or unmodified exendin-4 with the final concentration ranging from 10^-10^ to 10^-6^ M. The total volume was adjusted to 1 mL with medium (DMEM with 0.1% BSA) and the cells incubated on ice for 60 minutes. For the total binding no ^nat^In-labelled peptide was added. After incubation the cells were washed twice with phosphate buffered saline (PBS), solubilised with 1 mL 1 M sodium hydroxide (NaOH), collected and the activity quantified using a γ-counter (Packard Cobra II Auto Gamma, Perkin Elmer, Switzerland). The IC_50_ values were calculated by fitting the data with non-linear regression using least squares fit with GraphPad Prism (GraphPad Software, La Jolla, CA). Experiments were performed on triplicate samples. The statistical significance was determined using a one-way ANOVA test and corrected for multiple comparisons using Tukey’s honest significance test.

### Internalisation assay

The internalisation kinetics of ^111^In-PSI-CLNOD1, ^111^In-PSI-CLNOD2 and ^111^In-PSI-CLNOD3 were evaluated as described previously [26]. 4kBq (0.2–1 pmol) of each ^111^In-labelled peptide was used as a probe. After addition of the tracer the cells were incubated for certain time points (5 min, 15 min, 30 min, 60 min and 120 min, respectively) at 37°C; non-specific binding was determined by the addition of the corresponding ^nat^In-labelled probe to a final concentration of 1 μM. After incubation, the supernatant was aspirated and the cells washed with PBS. Both the supernatant and wash fractions were used to determine the non-bound fraction. In order to determine the peptide fraction bound on the surface the cells were incubated with 1 mL glycine buffer pH 2.6 for 5 min at room temperature and collected separately. The internalised fraction was identified by adding 1 mL 1M NaOH to the cells with subsequent collection of the lysates. The activity in all three fractions was measured in a γ-counter (Packard Cobra II Auto Gamma, Perkin Elmer, Switzerland). Experiments were performed on triplicate samples. The statistical significance was determined using the one-way ANOVA test and corrected for multiple comparisons using Tukey’s honest significance test.

### Plasma stability

Plasma stability was qualitatively determined as described previously [26]. 5 MBq (0.4 nmol) labelled peptide was added to fresh human blood plasma and incubated at 37°C for 48 h. Samples were taken before incubation as well as after 1 h, 4 h, 24 h and 48 h. Plasma proteins were precipitated using a solution containing 50% methanol and 50% acetonitrile with 0.1% TFA. The sample was then filtered through a Thomson Single StEP Filter vial 0.45μm PVDF (Thomson Instrument Company, Oceanside, CA) and analysed using RP-HPLC on a Discovery BioWide Pore C18 (15cm x 2.1mm; 3μm) column. The column was eluted with 95% water containing 0.1% TFA and 5% acetonitrile containing 0.1% TFA for 5 min followed with a linear gradient from 5 to 70% acetonitrile for 15 minutes succeeded by a second linear gradient from 70–90% acetonitrile for 5 min and an isocratic gradient at 95% acetonitrile for the final 5 min with a flow rate of 1 mL/min.

### Meprin β immunofluorescence staining

Immunofluorescence staining was performed on kidney cryosections obtained from CD1 nu/nu mice. The 3 μm thick sections were transferred onto a microscope slide, fixed in acetone for 10 min at 4°C and washed in PBS with 0.4% Triton X-100 (PBST) for 5 min. The sections were then treated with 1% donkey serum (Dako, Baar, Switzerland) in PBST, followed by blocking with 5% donkey serum in PBS for 1 h. Subsequently, the cells were incubated with polyclonal antibody directed against meprin β (sc-23491) (Santa Cruz Biotechnology, Santa Cruz, USA) diluted 1:200 in 5% donkey serum PBS for 1 h. After washing the sections with PBS they were incubated with polyclonal donkey anti-goat IgG H&L (Alexa Fluor 488) (ab150129) (Abcam, Cambridge, UK) diluted 1:600 in 5% donkey serum PBS for 1 h at room temperature. The sections were washed again and mounted with ProLong Gold antifade mountant (Life Technologies Europe B.V., Zug, Switzerland). Goat IgG, diluted 1:200 in 5% donkey serum PBS was used as primary control antibody. The fluorescent signal was visualised with a Leica SP5 Confocal Microscope (Leica Microsystems GmbH, Wetzlar, Germany) at 488 nm. The contrast of all pictures was increased to the same extent to improve the visibility of the signal.

### *In vitro* meprin digestion

Recombinant meprin α and meprin β were expressed in High Five Cells (BTI-TN-5B1-4) derived from the parental *Trichopulsia ni* cell line, purified and activated as described previously [27,28]. 220 ng of each recombinant protein was added to 35.5 μL 0.1 mM In-labelled peptide solution in 37.5 μL meprin assay buffer (50 mM Tris-HCl pH 7.5 + 1 mM MgCl_2_ + 1 mM CaCl_2_) and 30 μL 1 M Tris pH 7.5 and incubated at 37°C. Samples were taken at specific time points (0 min, 5 min, 10 min, 20 min and 60 min) and transferred into precipitation solution (methanol/acetonitrile with 0.1% TFA 1:1). The samples were centrifuged at 11’000 g for 2 min and qualitatively analysed using both RP-HPLC and liquid chromatography-mass spectrometry (LC-MS) on a Discovery Bio Wide Pore C18 (25 cm x 4.6 mm; 5 μm) column. The LC-MS samples were ionised using electrospray ionisation (ESI+) and the charged molecules detected using time of flight mode (TOF). The column was eluted with water (0–5 min) followed by a linear gradient from 0–95% acetonitrile for 40 min and an isocratic elution with 95% acetonitrile for the final 5 min at a flow rate of 1 mL/min for the whole run. For the LC-MS samples 0.1% formic acid was added to the eluents whereas for the RP-HPLC 0.1% TFA was supplemented.

### *Ex vivo* stability in kidney homogenates

Kidneys were extracted from CD1 nu/nu mice and shock-frozen in liquid nitrogen. The frozen tissue was crushed using a Bessman Tissue Pulveriser (Spectrum Chemical Manufacturing Corp., New Brunswick, NJ), transferred into a tube with Krebs-Henseleit buffer pH 7.4 and homogenised using a 20G syringe.

1.2 MBq (9.5–45.5 nmol) of either of the ^111^In-labelled peptides were added to the homogenate and incubated at 37°C. Samples were taken before incubation as well as after 30 min, 60 min and 180 min and mixed with precipitation solution (50% and 50% acetonitrile with 0.1% TFA). The samples were centrifuged twice at 11’000 g and qualitatively analysed via RP-HPLC.

### Biodistribution

Biodistribution of ^111^In-PSI-CLNOD1, ^111^In-PSI-CLNOD2, ^111^In-PSI-CLNOD3 and ^111^In-Ex4NOD40 were performed in CD 1 nu/nu mice with tumour xenografts that were prepared as previously described [26]. Six week-old female CD1 nu/nu mice were subcutaneously inoculated in both shoulder regions with 8 x 10^6^ CHL-GLP-1R positive cells suspended in PBS pH 7.4. After 4 weeks, the mice were randomly divided into groups of four mice. For the ^111^In-labelled fragments mice without tumours were used. The mice were injected with approximately 100 kBq (1–5 pmol) of the respective ^111^In-labelled peptide diluted in PBS pH 7.4 with 0.1% BSA via the tail vein and sacrificed after 4 h by CO_2_ asphyxiation. In order to determine the GLP-1R mediated uptake of ^111^In-PSI-CLNOD1, ^111^In-PSI-CLNOD2, ^111^In-PSI-CLNOD3 and ^111^In-Ex4NOD40 another group of four mice each were pre-injected with an excess of 100 μg exendin-4 to block the receptor and sacrificed after 4 h. Blood, heart, lungs, spleen, kidneys, pancreas, stomach, intestine, liver, muscle, bone and tumour were removed, weighted and activity determined in a γ-counter (Packard Cobra II Auto Gamma, Perkin Elmer, Switzerland). The percentage of injected activity per gram tissue (%iA/g) was calculated for each sample. The statistical significance was determined using the two-way ANOVA test and corrected for multiple comparisons using Tukey’s honest significance test. The data was log-transformed to account for the variance in the groups.

### Ethics Statement

All animal experiments were reviewed by the “Ethical Animal Committee of the Cantons Aarau, Baselland and Baselstadt), approved by the Cantonal Veterinarian Department of the Canton Aarau (permit number: 75531) and conducted in accordance with the Swiss law of animal protection.

## Results and Discussion

### Radiolabelling

All peptides were successfully labelled with ^111^In resulting in specific activities from 37.6 to 40.9 MBq/nmol. For the *in vitro* meprin α/β digestion the peptides were labelled with lower specific activities ranging from 0.07 to 0.25 MBq/nmol. In all cases the radiochemical yield was >95% as determined by RP-HPLC. ^111^In-DTPA eluted after 2 min while the ^111^In-labelled peptides were retained for approximately 8 to 9 min.

### IC_50_ binding assay

**Fig 3:**
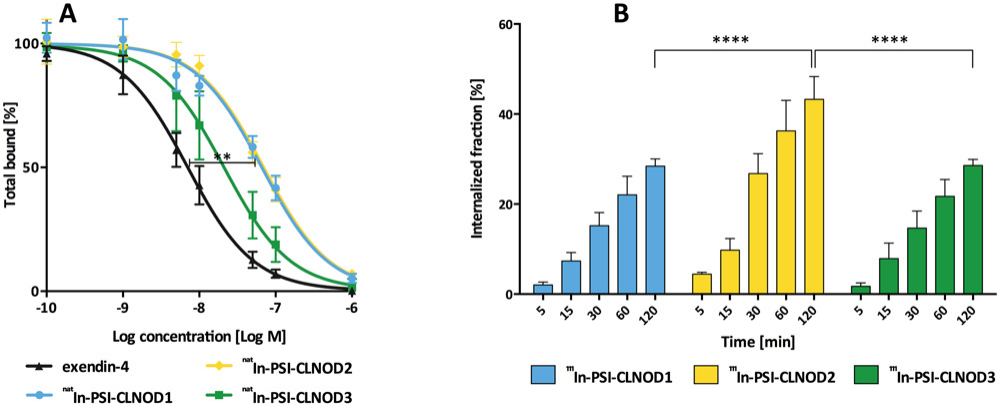
*In vitro* assessment of the peptides containing cleavable linkers in CHL cells expressing GLP-1R. (A) IC_50_ values of ^nat^In labelled peptides. 0.1–0.6 pmol ^111^In-Ex4NOD40 was used as tracer. (B) Internalisation kinetics of the ^111^In-labelled probes. 0.2–1 pmol of the respective ^111^In-labelled peptide was used as tracer. Non-specific binding was determined by incubation with 1 μM of ^nat^In labelled peptide.

In a competitive binding assay we showed that the ^nat^In-labelled peptides with the cleavable linkers retain a high affinity to the GLP-1R (Fig 3A). The binding of ^nat^In-PSI-CLNOD3, the peptide with the least modified amino acids, seems not to be impaired compared to unmodified exendin-4 as the difference is not significant (IC_50_^nat^In-PSI-CLNOD3: 21 nM, 95% confidence interval: 17 nM-26 nM; IC_50_ exendin-4: 7.0 nM, 95% confidence interval: 5.9 nM-8.3 nM). The other tested probes showed a significantly lower binding affinity (*p*<0.01) than exendin-4. The IC_50_ values of the other peptides were 66 nM (95% confidence interval 59–83 nM) for ^nat^In-PSI-CLNOD1 and 70 nM (95% confidence interval 60–81 nM) for ^nat^In-PSI-CLNOD2, respectively. Overall, all peptides have similar affinities as previously published peptides, which indicates that the C-terminal modification seems not to impair the binding affinity [26,29].

### Internalisation

All the ^111^In-labelled peptides internalise well, as seen in Fig 3B. Both ^111^In-PSI-CLNOD1 and ^111^In-PSI-CLNOD3 internalise over a similar timescale with approximately 28% uptake after 2 h, which is in the same range as already published peptides [26]. ^111^In-PSI-CLNOD2 on the other hand has a higher rate of internalisation with 43% of the peptide taken up into the cells after 2 h. The unspecific binding of PSI-CLNOD2, however, is also significantly higher (2.0 ± 0.8%) than for the other two peptides (^111^In-PSI-CLNOD1: 1.0 ± 0.2%; ^111^In- PSI-CLNOD3: 1.1 ± 0.7%) (p<0.05).

### Plasma stability

**Fig 4:**
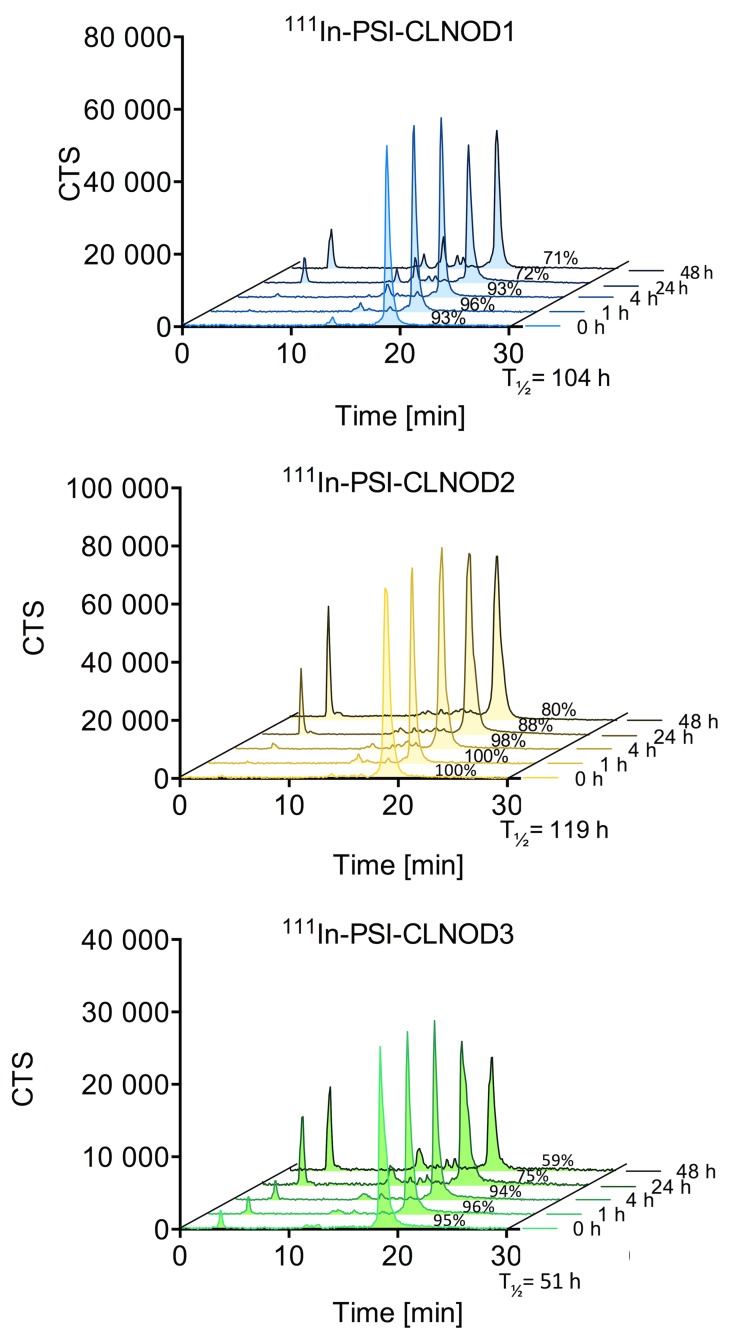
Plasma stability of ^111^In-labelled PSI-CLNOD1, PSI-CLNOD2 and PSI-CLNOD3 in fresh human blood plasma.

The plasma stability of the ^111^In-labelled peptides is illustrated in Fig 4. All peptides are >90% stable after 4 h. After 48 h 71% of PSI-CLNOD1, 80% of PSI-CLNOD2 and 59% of PSI-CLNOD3 are still intact. While PSI-CLNOD3 is less stable than previously tested peptides, all probes are >90% intact after 4 h, which is sufficient for SPECT imaging as, scans with ^111^In labeled exendin derivatives are usually performed after 4 h [22,26].

### *In vitro* meprin digestion

**Fig 5:**
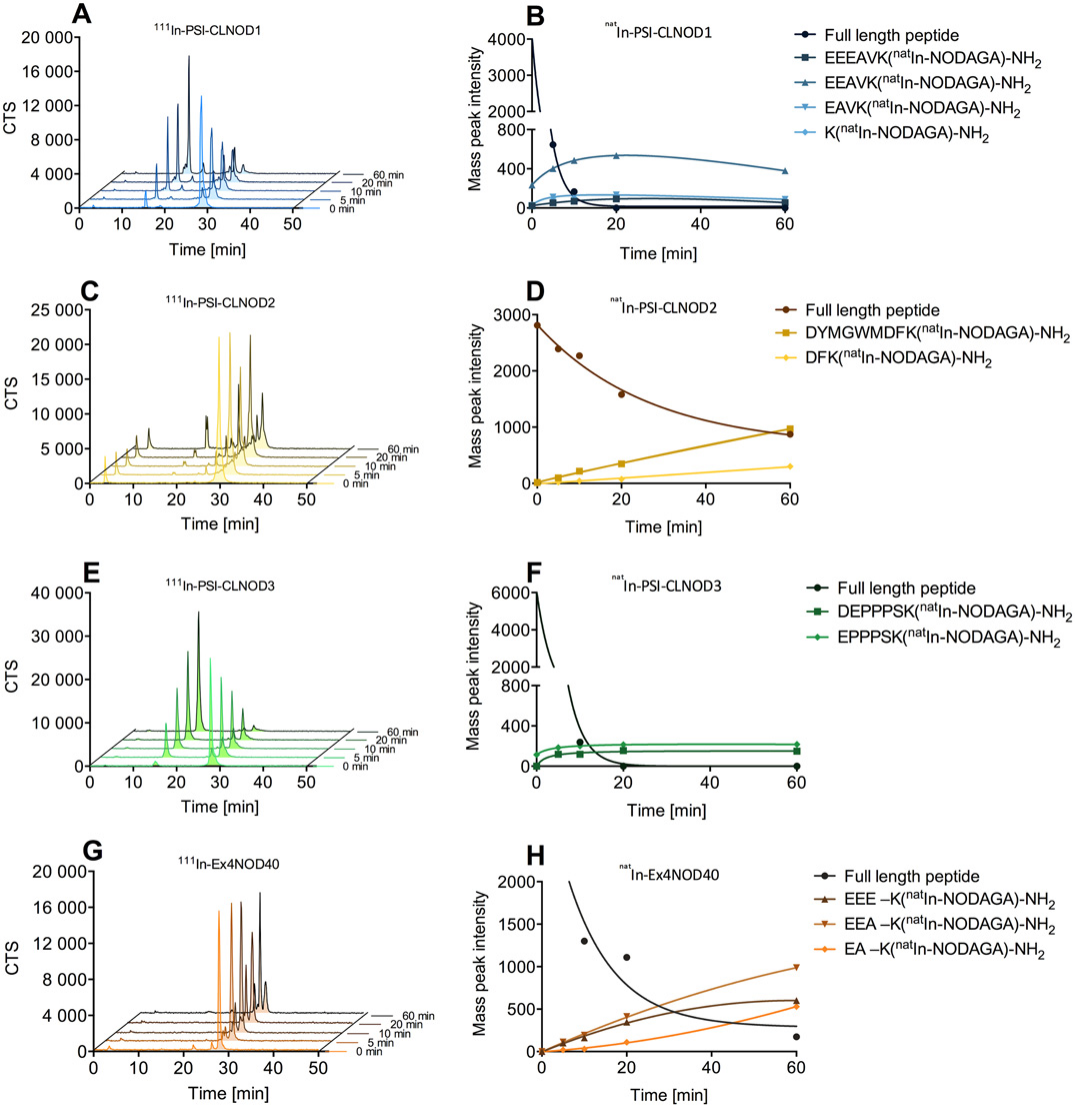
Digestion of In-labelled probes with recombinant meprin β. A, C, E, and G show the metabolism of the corresponding ^111^In-labelled peptides. Curves B, D, F and H show the degradation of the corresponding ^nat^In-labelled peptides and the formation of different fragments over time.

**Fig 6:**
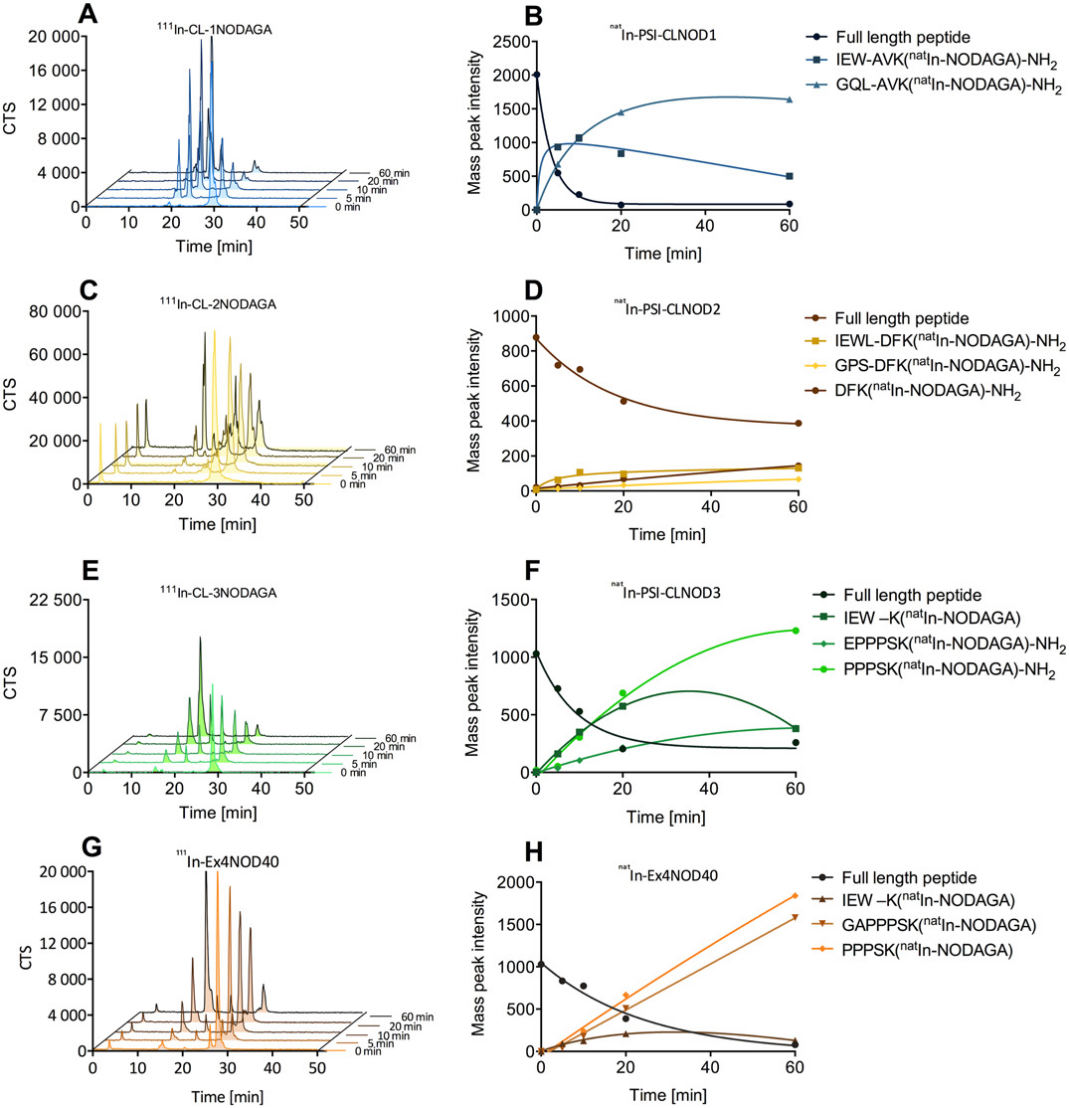
Digestion of In-labelled probes with recombinant meprin α. A, C, E, and G show the metabolism of the corresponding ^111^In-labelled peptides. Curves B, D, F and H show the degradation of the corresponding ^nat^In-labelled peptides and the formation of different fragments over time.

Figs 5 and 6 show the results of the digestion of the In-labelled probes with meprin α and meprin β, respectively. Both ^111^In-PSI-CLNOD1 and ^111^In-PSI-CLNOD3 are cleaved by meprin β and quickly form metabolites with retention times of approximately 15 min. Closer LC-MS analysis of the metabolites revealed four fragments for PSI-CLNOD1 and two fragments for PSI-CLNOD3, respectively, all of which were in the predicted region as seen in Fig 1. After 60 min only 7% of full length PSI-CLNOD1 and 3% of PSI-CLNOD3 remain intact. ^111^In-PSI-CLNOD2, in contrast is not cleaved completely as after 60 min approximately 20% of the full-length peptide remains in the sample.

All of the detected fragments were expected as the linkers were designed to be cleaved at specific positions [12]. The quick metabolism of both PSI-CLNOD1 and PSI-CLNOD3 might have been facilitated by a series of adjecent acidic amino acids that form a sequence recognised by meprin β, as seen in Fig 1, while PSI-CLNOD2 on the other hand is not metabolised as quickly as it contains only two, non-adjacent, aspartic acids. Furthermore, the localisation of one of the cleavage sites close to the chelator might sterically hinder the enzymatic digestion. The proximity to the chelator, however, is necessary as smaller fragmets are preferable since they are excreted more easily and rapidly.

Additionally, we tested the specificity of meprin α towards the cleavable linkers, although not as specific as meprin β but also expressed in the kidney. Meprin α digestion of the peptides also led to rapid breakdown of PSI-CLNOD1 and PSI-CLNOD3 with only approximately 6% of each peptide remaining intact after 60 min, while approximately 25% of PSI-CLNOD2 was intact 1 h after digestion. Cleavage sites for meprin α within the linker sequence were identified for both PSI-CLNOD2 and PSI-CLNOD3, for PSI-CLNOD1 cleavage was only found within the binding sites.

As meprin α lacks the same high specifictiy for certain amino acid sequences it is not surprising that those cleavage sites do not show a specific pattern [15]. Nevertheless, we were able to identify several sequences that are cleaved by meprin α, some of which are also recognised by meprin β (Table 2). ^111^In-Ex4NOD40 is also metabolised by both meprin α and meprin β. Cleavage by meprin β, however, yields larger fragments that are more likely to be retained in the kidneys. Multiple cleavage sites for meprin β were identified between M14 and E17 while cleavage by meprin α was found at F22, S33 and A35.

**Table 2:** Cleavage sites of In-labelled peptides digested with meprin α and β.

**^nat^In-PSI-CLNOD1**	HGEGTFTSDLSKQNleEEEAVRLFIEWLKNGGPSSG**↑QNle** **↓** **E** **↓** **E** **↓** **EAV** **↓**K(^nat^In-NODAGA)-NH_2_
**^nat^In-PSI-CLNOD2**	HGEGTFTSDLSKQNleEEEAVRLFIEWLKNG↑GPSSG↓**DYMGWM↑↓DF**K(^nat^In-NODAGA)-NH_2_
**^nat^In-PSI-CLNOD3**	HGEGTFTSDLSKQNleEEEAVRLFIEWLKNGGPS**E**↓↑**D**↑↓**E**PPPSK(^nat^In-NODAGA)-NH_2_
**^nat^In-Ex4NOD40**	HGEGTFTSDLSKQM↓E↓E↓EAVRLF↑IEWLKNGGPSS↑GA↑PPPSK(^nat^In-NODAGA)-NH_2_

Cleavage sites for meprin α are illustrated with ↑, for meprin β with ↓. The linkers are highlighted in bold. In-labelled Ex4NOD40 was used as control.

### Meprin β immunofluorescence staining

**Fig 7:**
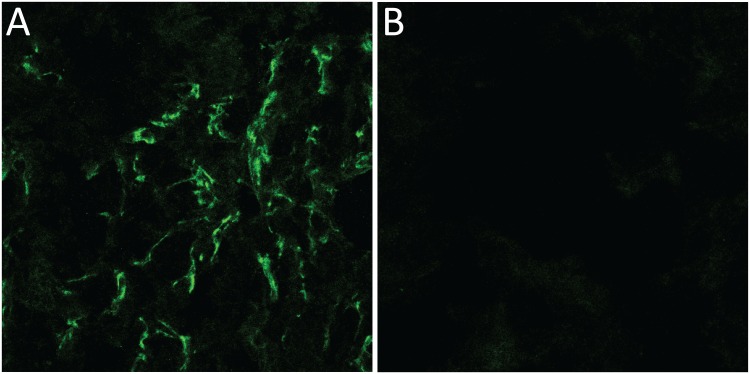
Immunofluorescence picture of murine kidney sections. (A) Anti-Meprin β antibody was used to detect the protease, visualised by fluorescence detection at 488 nm. (B) For the control goat igG was used.

To verify the expression of meprin β in the kidneys we performed immunolfuorescence staining on kidney sections. Fig 7 shows a clear flourescent signal originating from the brush-border membrane confirming the viability of the target.

### *Ex vivo* stability in kidney homogenates

**Fig 8:**
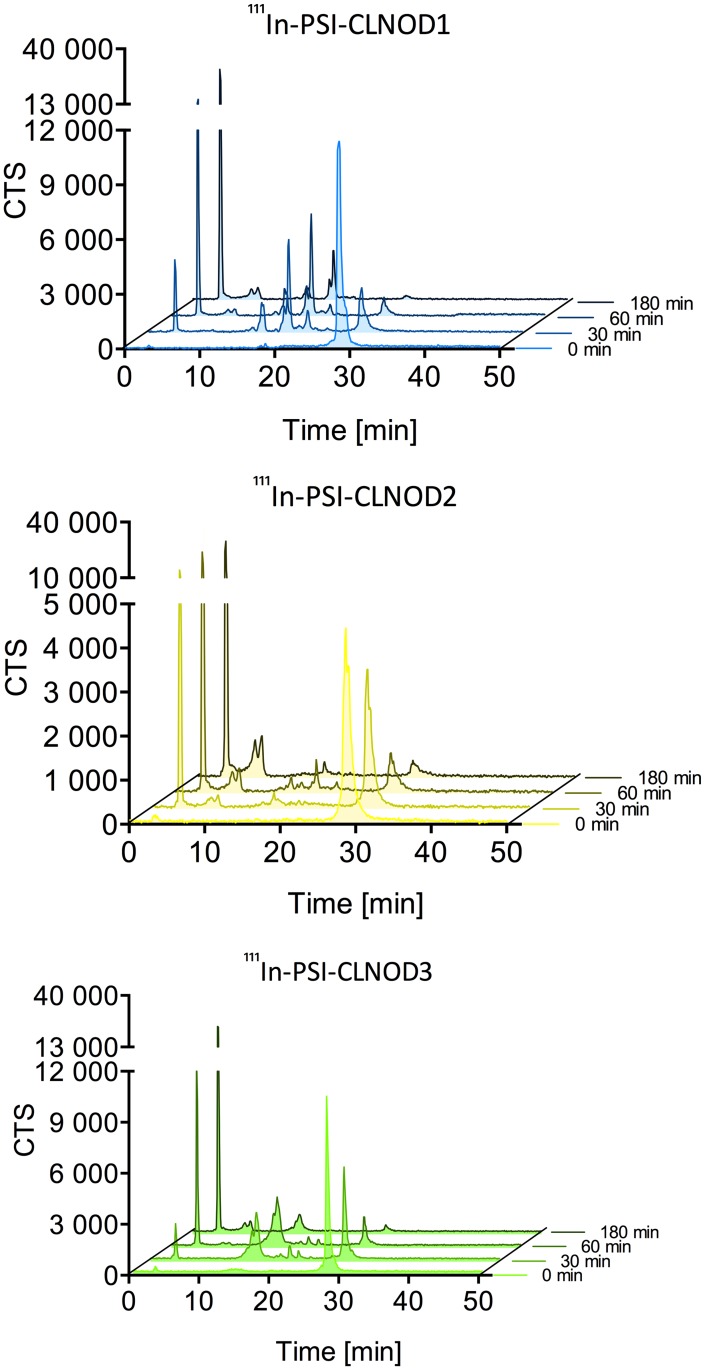
*Ex vivo* metabolism of the corresponding peptides in CD1 nu/nu kidney homogenates.

All the ^111^In-labelled peptides with cleavable linkers are metabolised quickly after incubation with CD1 kidney homogenates. Fragments with similar retention-times as after the meprin β digestion are formed within 30 min, as seen in Fig 8. One metabolite not present in the meprin β digestion, however, is formed and elutes after 3 minutes, indicating a small, very polar fragment. This fragment is formed in all peptides with cleavable linkers and is the only remaining peak after 180 min for ^111^In-PSI-CLNOD3 and the major peak for both ^111^In-PSI-CLNOD1 and ^111^In-PSI-CLNOD2. Radiolabelled Ex4NOD40 showed a similar pattern as within 30 min the majority of the peptide was digested leaving the very polar fragment described above. These additional fragments are most likely a product of unspecific cleavage of the linkers by other proteases that are present in the homogenate and potentially the brush border membrane.

### Biodistribution

**Fig 9:**
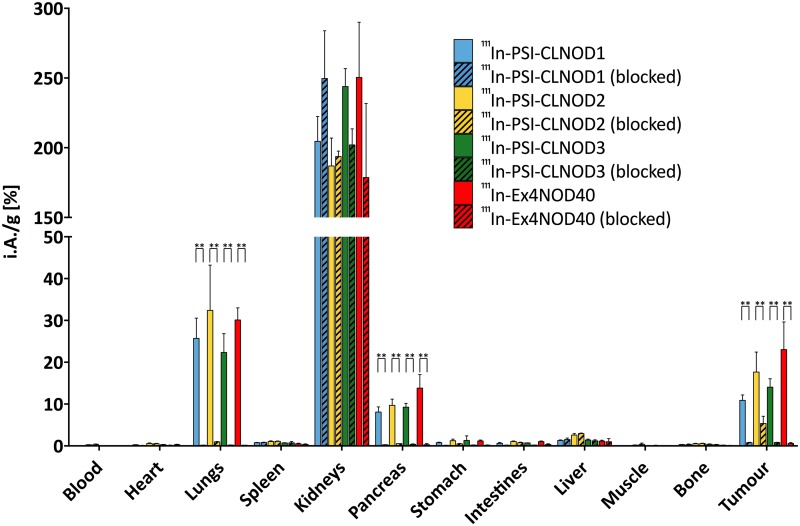
Biodistribution of ^111^In-labeled peptides in CD1 nu/nu mice with CHL-GLP1R positive tumour-xenografts. Values are mean percentages injected dose per gram tissue (*n* = 4. Error bars SD). Blocking was performed by pre-injection of 100 μg excess of unmodified exendin-4. Mice were sacrificed 4 h after injection. The significance was tested by a two-was ANOVA test corrected for multiple comparisons by Tukey’s honest significance test (**p<0.01; ***p<0.001; ****p>0.0001).

^111^In-labelled PSI-CLNOD1, PSI-CLNOD2 and PSI-CLNOD3 as well as Ex4NOD40 were specifically taken up in the receptor-positive tissues as tumour, pancreas and lungs of CD1 nude mice 4 h after administration. The uptake in those organs was blocked by the pre-injection of 100 μg exendin-4 (p<0.01). Out of the three peptides with the cleavable linkers ^111^In-PSI-CLNOD2 displayed the highest uptake in the tumour, its unspecific uptake in the receptor positive tissues, however, was also the highest (p<0.05), which correlates with the results from the internalisation assay (Fig 9 and S2 Table). All of ^111^In-labelled peptides with the cleavable linkers had comparable uptake in GLP-1R positive tissue and were not significantly different from the uptake of the lead compound. Overall, the uptake in receptor positive tissue is comparable to already published radiolabelled exendin-4 derivatives [26].

The most activity in all four cases, however, accumulates in the kidneys. It was shown previously that this accumulation is mainly by non-GLP-1R mediated uptake and is primarily facilitated by various other mechanisms including the Megalin transporter [26,29–32]. None of the ^111^In-labelled peptides show a significant difference in kidney retention compared to the lead compound ^111^In-Ex4NOD40. A biodistribution of the ^111^In-labelled fragments CL1F1, CL1F2, CL1F3, CL2F1, CL2F2, CL3F1 and CL3F2 we identified after meprin β cleavage of the peptides revealed that the fragments that are formed after digestion are not retained in the kidneys as less than 4% of the injected activity per gram tissue were detected at the end of the experiment (S3 Table). This leads to the assumption that the peptides are not cleaved *in vivo* before being reabsorbed, either because the expression of meprin in the brush border membrane is too low to digest the linkers quickly enough or because the rate of the re-uptake mechanism of exendin-4 exceeds the turnover rate of meprin.

## Conclusions

In conclusion, we designed three new exendin-4 derivatives containing specific cleavage sites for meprin β. ^111^In-labelled PSI-CLNOD1, PSI-CLNOD2 and PSI-CLNOD3 retain the excellent *in vitro* properties of previously tested exendin-4 derivatives as well as the high specific uptake into GLP-1R positive tissue. This suggests that the seven C-terminal amino acids may be altered without loss of affinity towards the GLP-1R receptor, allowing new strategies of modifying exendin-4 to be developed. Uptake of the peptides into the kidneys, however, was not decreased even though we could show that meprin β is expressed in the brush border membrane, leading to the assumption that the uptake mechanism of the peptides is faster than the cleavage of the linker. The rapid and specific cleavage by meprin β, and to a lesser extent by meprin α *in vitro*, however, demonstrates the potential of the concept of specific linkers for meprin targeting as prodrugs in conditions where meprin β expression is upregulated.

## Supporting Information

S1 Table Amino acid sequences of the peptides used in this work. (DOCX)

S2 Table Biodistribution of ^111^In-labelled peptides in CD1 nu/nu mice with CHL-GLP1R positive tumour xenografts. Values are mean percentages injected dose per gram tissue (*n* = 4. Error bars SD). Blocking was performed by pre-injection of 100 μg excess of unmodified exendin-4. Mice were sacrificed 4 h after injection.
(DOCX)

S3 Table Biodistribution of the ^111^Inlabelled fragments of the corresponding peptides in CD1 nu/nu mice. (DOCX)
