# Supplementary material for: Evaluation of ¹¹¹In-Labelled Exendin-4 Derivatives Containing Different Meprin β-Specific Cleavable Linkers
Source: PLoS One. 2015 Apr 9;10(4):e0123443. doi: 10.1371/journal.pone.0123443 (PMC4391719; doi:10.1371/journal.pone.0123443)
Supplement: S1 Table — (DOCX) [file pone.0123443.s001.docx]

**Supporting information:**

Table S1 Amino acid sequences of the peptides used in this work.

| Ex4NOD40 | HGEGTFTSDLSKQMEEEAVRLFIEWLKNGGPSSGAPPPSK(NODAGA)-NH_2_ |
| --- | --- |
| PSI-CLNOD1 | HGEGTFTSDLSKQNleEEEAVRLFIEWLKNGGPSSGQNleEEEAVK(NODAGA)-NH_2_ |
| PSI-CLNOD2 | HGEGTFTSDLSKQNleEEEAVRLFIEWLKNGGPSSGDYMGWMDFK(NODAGA)-NH_2_ |
| PSI-CLNOD3 | HGEGTFTSDLSKQNleEEEAVRLFIEWLKNGGPSEDEPPPSK(NODAGA)-NH_2_ |
