# Supplementary material for: Evaluation of ¹¹¹In-Labelled Exendin-4 Derivatives Containing Different Meprin β-Specific Cleavable Linkers
Source: PLoS One. 2015 Apr 9;10(4):e0123443. doi: 10.1371/journal.pone.0123443 (PMC4391719; doi:10.1371/journal.pone.0123443)
Supplement: S2 Table — Values are mean percentages injected dose per gram tissue (n = 4. Error bars SD). Blocking was performed by pre-injection of 100 μg excess of unmodified exendin-4. Mice were sacrificed 4 h after injection. (DOCX) [file pone.0123443.s002.docx]

**Supporting information:**

Table S2: Biodistribution of ^111^In-labelled peptides in CD1 nu/nu mice with CHL-GLP1R positive tumour xenografts. Values are mean percentages injected dose per gram tissue (n = 4. Error bars SD). Blocking was performed by pre-injection of 100 μg excess of unmodified exendin-4. Mice were sacrificed 4 h after injection.

| Biodistribution (%iA/g) | | | | | | |
| --- | --- | --- | --- | --- | --- | --- |
|  | **4 h** | | | **4 h (blocked)** | | |
|  | **PSI-CLNOD1** | | | | | |
| Blood | 0.05 | ± | 0.01 | 0.04 | ± | 0.00 |
| Heart | 0.27 | ± | 0.03 | 0.12 | ± | 0.02 |
| Lungs | 25.69 | ± | 4.85 | 0.18 | ± | 0.03 |
| Spleen | 0.77 | ± | 0.06 | 0.81 | ± | 0.08 |
| Kidneys | 204.57 | ± | 17.86 | 249.69 | ± | 34.24 |
| Pancreas | 8.10 | ± | 1.22 | 0.26 | ± | 0.04 |
| Stomach | 0.76 | ± | 0.15 | 0.15 | ± | 0.03 |
| Intestines | 0.62 | ± | 0.17 | 0.17 | ± | 0.03 |
| Liver | 1.32 | ± | 0.12 | 1.53 | ± | 0.33 |
| Muscle | 0.09 | ± | 0.03 | 0.07 | ± | 0.02 |
| Bone | 0.32 | ± | 0.05 | 0.33 | ± | 0.14 |
| Tumour | 10.91 | ± | 1.29 | 0.78 | ± | 0.08 |
|  | **PSI-CLNOD2** | | | | | |
| Blood | 0.25 | ± | 0.04 | 0.39 | ± | 0.08 |
| Heart | 0.59 | ± | 0.11 | 0.52 | ± | 0.08 |
| Lungs | 32.39 | ± | 10.82 | 0.97 | ± | 0.07 |
| Spleen | 1.04 | ± | 0.17 | 1.09 | ± | 0.09 |
| Kidneys | 186.90 | ± | 20.04 | 193.69 | ± | 3.92 |
| Pancreas | 9.70 | ± | 1.48 | 0.54 | ± | 0.01 |
| Stomach | 1.24 | ± | 0.36 | 0.49 | ± | 0.11 |
| Intestines | 1.06 | ± | 0.12 | 0.76 | ± | 0.15 |
| Liver | 2.60 | ± | 0.34 | 2.94 | ± | 0.11 |
| Muscle | 0.17 | ± | 0.07 | 0.38 | ± | 0.28 |
| Bone | 0.53 | ± | 0.08 | 0.59 | ± | 0.06 |
| Tumour | 17.65 | ± | 4.78 | 5.35 | ± | 1.74 |
|  | **PSI-CLNOD3** | | | | | |
| Blood | 0.07 | ± | 0.01 | 0.05 | ± | 0.00 |
| Heart | 0.31 | ± | 0.04 | 0.16 | ± | 0.03 |
| Lungs | 22.35 | ± | 4.49 | 0.21 | ± | 0.02 |
| Spleen | 0.72 | ± | 0.05 | 0.75 | ± | 0.31 |
| Kidneys | 243.91 | ± | 12.82 | 202.04 | ± | 11.49 |
| Pancreas | 9.27 | ± | 0.90 | 0.35 | ± | 0.14 |
| Stomach | 1.32 | ± | 1.09 | 0.21 | ± | 0.04 |
| Intestines | 0.69 | ± | 0.03 | 0.19 | ± | 0.02 |
| Liver | 1.39 | ± | 0.22 | 1.22 | ± | 0.23 |
| Muscle | 0.09 | ± | 0.02 | 0.11 | ± | 0.07 |
| Bone | 0.40 | ± | 0.08 | 0.29 | ± | 0.07 |
| Tumour | 14.03 | ± | 2.04 | 0.79 | ± | 0.07 |
|  | **Ex4NOD40** | | | | | |
| Blood | 0.06 | ± | 0.01 | 0.03 | ± | 0.01 |
| Heart | 0.29 | ± | 0.10 | 0.05 | ± | 0.00 |
| Lungs | 30.10 | ± | 2.89 | 0.16 | ± | 0.02 |
| Spleen | 0.52 | ± | 0.11 | 0.36 | ± | 0.14 |
| Kidneys | 250.38 | ± | 39.62 | 178.63 | ± | 53.16 |
| Pancreas | 13.83 | ± | 3.23 | 0.31 | ± | 0.29 |
| Stomach | 1.17 | ± | 0.22 | 0.18 | ± | 0.06 |
| Intestines | 1.04 | ± | 0.16 | 0.34 | ± | 0.17 |
| Liver | 1.15 | ± | 0.21 | 0.98 | ± | 0.76 |
| Muscle | 0.07 | ± | 0.04 | 0.08 | ± | 0.03 |
| Bone | 0.16 | ± | 0.03 | 0.04 | ± | 0.10 |
| Tumour | 23.03 | ± | 6.60 | 0.58 | ± | 0.22 |
