# Supplementary material for: Evaluation of ¹¹¹In-Labelled Exendin-4 Derivatives Containing Different Meprin β-Specific Cleavable Linkers
Source: PLoS One. 2015 Apr 9;10(4):e0123443. doi: 10.1371/journal.pone.0123443 (PMC4391719; doi:10.1371/journal.pone.0123443)
Supplement: S3 Table — (DOCX) [file pone.0123443.s003.docx]

**Supporting information:**

Table S3: Biodistribution of the ^111^Inlabelled fragments of the corresponding peptides in CD1 nu/nu mice.

| Biodistribution (%iA/g) after 4 h | | | | | | | | | |
| --- | --- | --- | --- | --- | --- | --- | --- | --- | --- |
|  | **PSI-CLNOD1** | | | | | | | | |
|  | **CL1-F1** | | | **CL1-F2** | | | **CL1-F3** | | |
| Blood | 0.01 | ± | 0.01 | 0.01 | ± | 0.00 | 0.00 | ± | 0.00 |
| Heart | 0.01 | ± | 0.01 | 0.02 | ± | 0.01 | 0.01 | ± | 0.00 |
| Lungs | 0.03 | ± | 0.00 | 0.04 | ± | 0.02 | 0.03 | ± | 0.02 |
| Spleen | 0.03 | ± | 0.01 | 0.03 | ± | 0.01 | 0.03 | ± | 0.00 |
| Kidneys | 2.28 | ± | 0.47 | 2.97 | ± | 0.75 | 1.72 | ± | 0.28 |
| Pancreas | 0.01 | ± | 0.01 | 0.01 | ± | 0.00 | 0.01 | ± | 0.00 |
| Stomach | 0.02 | ± | 0.01 | 0.02 | ± | 0.01 | 0.02 | ± | 0.01 |
| Intestines | 0.22 | ± | 0.12 | 0.10 | ± | 0.02 | 0.09 | ± | 0.03 |
| Liver | 0.07 | ± | 0.01 | 0.08 | ± | 0.00 | 0.08 | ± | 0.01 |
| Muscle | 0.00 | ± | 0.01 | 0.00 | ± | 0.01 | 0.00 | ± | 0.01 |
| Bone | 0.05 | ± | 0.01 | 0.06 | ± | 0.03 | 0.07 | ± | 0.04 |
|  | **PSI-CLNOD2** | | | | | | | | |
|  | **CL2-F1** | | | **CL2-F2** | | |  |  |  |
| Blood | 0.02 | ± | 0.01 | 0.01 | ± | 0.00 |  |  |  |
| Heart | 0.02 | ± | 0.01 | 0.01 | ± | 0.00 |  |  |  |
| Lungs | 0.04 | ± | 0.01 | 0.03 | ± | 0.01 |  |  |  |
| Spleen | 0.07 | ± | 0.01 | 0.04 | ± | 0.01 |  |  |  |
| Kidneys | 2.50 | ± | 0.21 | 2.11 | ± | 0.30 |  |  |  |
| Pancreas | 0.02 | ± | 0.01 | 0.02 | ± | 0.01 |  |  |  |
| Stomach | 0.04 | ± | 0.01 | 0.03 | ± | 0.01 |  |  |  |
| Intestines | 0.15 | ± | 0.06 | 0.14 | ± | 0.12 |  |  |  |
| Liver | 0.17 | ± | 0.02 | 0.11 | ± | 0.03 |  |  |  |
| Muscle | 0.00 | ± | 0.01 | 0.01 | ± | 0.01 |  |  |  |
| Bone | 0.06 | ± | 0.02 | 0.01 | ± | 0.01 |  |  |  |
| PSI-CLNOD3 | | | | | | | | | |
|  | **CL3-F1** | | | **CL3-F2** | | |  |  |  |
| Blood | 0.01 | ± | 0.00 | 0.02 | ± | 0.01 |  |  |  |
| Heart | 0.00 | ± | 0.01 | 0.01 | ± | 0.00 |  |  |  |
| Lungs | 0.02 | ± | 0.01 | 0.04 | ± | 0.02 |  |  |  |
| Spleen | 0.01 | ± | 0.01 | 0.03 | ± | 0.01 |  |  |  |
| Kidneys | 1.71 | ± | 0.17 | 2.22 | ± | 0.50 |  |  |  |
| Pancreas | 0.00 | ± | 0.00 | 0.01 | ± | 0.00 |  |  |  |
| Stomach | 0.01 | ± | 0.00 | 0.02 | ± | 0.01 |  |  |  |
| Intestines | 0.04 | ± | 0.03 | 0.10 | ± | 0.11 |  |  |  |
| Liver | 0.04 | ± | 0.00 | 0.07 | ± | 0.02 |  |  |  |
| Muscle | -0.01 | ± | 0.01 | 0.02 | ± | 0.01 |  |  |  |
| Bone | -0.06 | ± | 0.04 | 0.21 | ± | 0.32 |  |  |  |
